# Using microRNA Networks to Understand Cancer

**DOI:** 10.3390/ijms19071871

**Published:** 2018-06-26

**Authors:** Mihnea Dragomir, Ana Carolina P. Mafra, Sandra M. G. Dias, Catalin Vasilescu, George A. Calin

**Affiliations:** 1 Department of Experimental Therapeutics, The University of Texas MD Anderson Cancer Center, 1515 Holcombe Blvd. Unit 1950, Houston, TX 77030, USA; mihnea.p.dragomir@gmail.com (M.D.); carolina.mafra@lnbio.cnpem.br (A.C.P.M.); 2 Department of Surgery, Fundeni Hospital, University of Medicine and Pharmacy Carol Davila, Sos. Fundeni nr. 258, Sector 2, 022328 Bucharest, Romania; 3 Research Center for Functional Genomics, Biomedicine and Translational Medicine, University of Medicine and Pharmacy Iuliu Hatieganu, Str. Gh. Marinescu 23, 400012 Cluj-Napoca, Romania; 4 Brazilian Biosciences National Laboratory (LNBio), Brazilian Center for Research in Energy and Materials (CNPEM), Rua Giuseppe Maximo Scolfaro 10000, Campinas, SP 13083-970, Brazil; sandra.dias@lnbio.cnpem.br; 5 Department of Genetics, Evolution and Bioagents, Institute of Biology, P.O. Box 6109, University of Campinas—UNICAMP, Campinas, SP 13083-970, Brazil; 6 Center for RNA Inference and Non-Coding RNAs, The University of Texas MD Anderson Cancer Center, 1515 Holcombe Blvd. Unit 1950, Houston, TX 77030, USA

**Keywords:** microRNAs, gene regulatory networks, neoplasms, molecular targeted therapy

## Abstract

Human cancers are characterized by deregulated expression of multiple microRNAs (miRNAs), involved in essential pathways that confer the malignant cells their tumorigenic potential. Each miRNA can regulate hundreds of messenger RNAs (mRNAs), while various miRNAs can control the same mRNA. Additionally, many miRNAs regulate and are regulated by other species of non-coding RNAs, such as circular RNAs (circRNAs) and long non-coding RNAs (lncRNAs). For this reason, it is extremely difficult to predict, study, and analyze the precise role of a single miRNA involved in human cancer, considering the complexity of its connections. Focusing on a single miRNA molecule represents a limited approach. Additional information could come from network analysis, which has become a common tool in the biological field to better understand molecular interactions. In this review, we focus on the main types of networks (monopartite, association networks and bipartite) used for analyzing biological data related to miRNA function. We briefly present the important steps to take when generating networks, illustrating the theory with published examples and with future perspectives of how this approach can help to better select miRNAs that can be therapeutically targeted in cancer.

## 1. Introduction

Cancer is a complex disease. Besides having a common set of traits, defined as the hallmarks of cancer [1], each tumor has its own singularities. Cancer is not a single disease alone; it comprises a series of histotypes with their own characteristics, with a combinatorial effect that culminates in and contributes to the diverseness of cancer. Because of its remarkable diversity, neoplastic disease has been one of the most difficult diseases to be researched and understood by modern science.

During the 1970s, Francis Crick asserted what he believed to be the central dogma of molecular biology. Genetic information traveled from DNA to RNA through transcription, then from RNA to proteins via translation, meaning proteins were the functional end products of genes [2]. However, after whole human genome sequencing, it was understood that genes that encoded proteins accounted for less than 2% of the genome [3]. Given the intricacy of cellular processes, genetic information is most likely passed by additional regulatory elements and not only by coding genes. 

Notwithstanding, it is now known that epigenetics plays an important role in gene expression regulation. Processes such as DNA methylation, histone modifications and non-coding RNA-mediated gene expression regulation are among those that are mainly described in this field (reviewed by [4]). The first non-coding RNAs (ncRNAs) described were lineage defective 4 (lin-4) [5] and lethal 7 (let-7) [6], proven to be essential during embryonic development of worms, despite their lack of protein-coding regions. It took as long as a decade until more studies uncovered a series of ncRNA species, constituting close to 60 to 70% of the transcriptional output in human cells [7].

Highly conserved throughout species [8,9,10], and of particular importance to this review, microRNAs (miRNAs) were found to be a family of small ncRNAs that regulate a variety of biological processes [11]. To date, there are almost 2000 annotated miRNA precursor genes [12], most of them having a general regulatory function across the genome through post-transcriptional regulation of messenger RNAs (mRNAs).

Usually, miRNAs do not only target one but several mRNAs, sometimes related to cellular function. However, most approaches regarding the study of miRNAs focus on identifying targets of a specific miRNA, as if its regulatory function occurs in isolation. However, it has been proven that each miRNA can regulate hundreds, or even thousands of mRNAs, while various miRNAs can control the same mRNA [13,14]. As a matter of fact, most mRNAs in the mammalian genome are targets of miRNAs [15], and many miRNAs regulate and are regulated by other species of ncRNAs, such as circular RNAs (circRNAs) [16,17] and long non-coding RNAs (lncRNAs) [18,19]. Therefore, it is hard to predict the regulatory functions of miRNAs by studying them separately. However, it is more likely that miRNAs naturally build regulatory networks that control different cellular functions [20,21,22].

In fact, Victor Ambros, in one of the first reviews regarding the regulatory functions of miRNAs, stated that “The identification of the regulatory targets of miRNAs (…) will probably require a combination of informatics, biochemical, and genetic approaches”. He predicted that “informatics approaches should be used to cast a broad net” in identifying functional miRNA and target interactions, supported by “validation using biochemical evidence of in vivo interactions and genetic epistasis” [23]. Thus, since the beginning of the miRNA study field, it was already recognized that miRNAs and their interactions were key regulators of the cellular program and machinery.

Not surprisingly, miRNAs were found to be involved in cancer pathogenesis and progression (reviewed by [24,25]). MiRNAs have been reported to be dysregulated in cancer [26], functioning at times as oncogenic (oncomiRs), or as having a tumor suppressive role [27]. In fact, the most studied miRNAs to date have shown to have multiple functions in cancer biology. 

For example, the deletion of the miR-15a/16-1 cluster and the primary transcript Leu-2 in chromosome 13 in B-cells of chronic lymphocytic leukemia (CLL) patients allows higher expression of its target, BCL2, an anti-apoptotic protein [26,28]. Contrarily, amplification of the miR-17-92 cluster in B-cell lymphomas and lung cancer leads to the overexpression of these miRNAs, associated with tumorigenesis [29,30]. In glioblastomas, p27 is a direct target of miR-221/222, promoting aggressive growth of these tumors [31]. *RAS*, the most frequently mutated oncogene in several types of cancers, was found to be regulated by the let-7 miRNA family in lung cancers [32]. Also, overexpression of miR-21 is associated with poorer prognosis in lung cancer, since it directly targets the Ras/MEK/ERK pathway [33]. Also, interestingly, Epstein-Barr virus (EBV) infection in malignant B-cells induces the expression of miR-21, potentially exacerbating the oncogenic function of miR-21 and the progression of plasmacytoid tumors [34]. MiR-34 acts in concert with other players of the p53 regulatory axis to block sustained proliferation of cancer cells [35]. Also with a tumor suppressive role, the miR-200 family acts during the epithelial-to-mesenchymal transition (EMT) process of invasion and metastasis, targeting one of its main transcription factors, ZEB1 [36], and also by indirectly up-regulating E-cadherin expression [37]. Interestingly, miRNAs have also a role in regulating the expression of checkpoint receptors, recently identified as very important targets for cancer immunotherapy. A recent study identified miR-200c and miR-34a as key regulators of programmed death-ligand 1(PD-L1), one of the most studied checkpoint receptors [38]. In this case, specifically, the oncoprotein mucin-1 (MUC1), when silenced, led to an increase in the level of these miRNAs, which in turn suppressed the expression of PD-L1 in acute myeloid leukemia (AML). Notwithstanding, when analyzing a network of miRNAs regulating checkpoint receptors (reviewed by [39]), it was found that various miRNAs can target multiple receptors, mimicking the effect of a combined immune checkpoint blockade. 

Because of their stronger phenotype induction, some of these miRNAs have made it through all clinical trials, acting as either mimic or antagomiRs, depending on their function as tumor suppressors or oncogenes, respectively. Unfortunately, miRNA therapies have not been as successful as expected in the clinic [40]. One of the reasons, among many factors involved, is that miRNAs do not have single targets. 

Besides, as being part of complex regulatory networks, many miRNAs have a context-dependent role in cancer (reviewed by [41]). This means that the same miRNAs might have a dual function, as oncogenes or tumor suppressors, depending on a number of factors, such as cancer and cell type, tissue location, cell location, etc. To add complexity, there are miRNA sponges, such as lncRNAs, pseudogenes and circRNAs, that bind to miRNAs and indirectly determine the overexpression of the mRNAs [42], termed competitive endogenous RNAs (ceRNAs) ([43]; reviewed by [44]). After discovering that CDR1as have over 70 miR-7 binding sites [16,17], circRNAs seemed to be the ideal sponges for miRNAs, confirming the ceRNA theory. Subsequently, it was shown that only few circRNAs display enough miRNA response elements (MREs) to alter the expression of miRNAs [45].

Combining the complexity of initiation, progression and metastasis of cancers with the intricate structure of connections in the regulatory functions of miRNAs, it is extremely difficult to predict, study and analyze the precise role of miRNAs in human cancer, especially if focusing on a single miRNA. Hence, we find ourselves working with this extremely versatile class of molecules, miRNAs, involved in a complex disease, cancer, and we are stuck in a maze of multiple targets that have to be functionally characterized in connection to each other. Ariadne’s thread of this dilemma could come from mathematical approaches of network analysis that have been extrapolated to the biological field to better understand molecular interactions. 

A highly used systems biology approach, network analysis could be the key to understand the cell’s functional organization [46,47]. In this review, we focus on the main types of networks for analyzing biological data (Table 1), briefly delineating important steps to take when drawing networks, illustrating the theory with published examples and with future perspectives of research in the field.

## 2. Understanding and Building miRNA Networks

**Figure 1:**
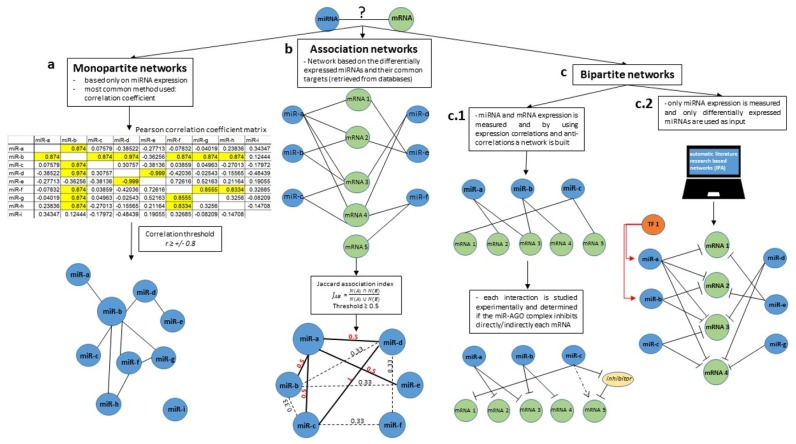
Building miRNA networks. The first questions that need to be answered before building a miRNA network are—what are the nodes? And what does an edge between two nodes mean? The different answers to these questions lead to the multiple types of miRNA networks that can be built. The network can be composed only of miRNA nodes. This type of networks containing only a single species of nodes are termed monopartite networks (**a**). The entire construct is based only on the expression level of miRNAs. The most common method employed to build monopartite graphs is the correlation coefficient. A correlation matrix is built using the expression level of several miRNAs in multiple patients of the same cohort. The entire method depends on choosing a correlation threshold. All the miRNAs from the matrix that have a higher correlation value than the threshold will be joined by an edge in the network construction. Therefore, in this case, an edge means a high correlation level between two miRNAs. A second option is to build association networks (**b**), which are composed of two types of nodes, usually miRNAs (differentially expressed) and their common mRNA targets based on available predicted/validated miRNA-mRNA interaction data. The next step in building association networks is the use of an association index (Jaccard, Simpson, Geometric and Cosine) that calculates the amount of shared targets between two miRNAs. Again, a threshold must be selected to define which miRNA nodes are interconnected and the final network is composed only of miRNA nodes and an edge is a measure of shared targets between two miRNAs. Finally, a last type of miRNA networks commonly found in literature is the bipartite network (**c**). This network contains two species of nodes: miRNAs and most commonly mRNAs. (**c.1**) One possible way to generate this type of network is by experimentally determining the expression of miRNAs and mRNAs and determine the anti-correlation between them. If two elements of different species anti-correlate/correlate above a given threshold, an edge can be drawn between these elements. (**c.2**) A second way to generate bipartite networks is by imputing the differentially expressed miRNAs in computer software (most commonly IPA) which generates, based on available data from literature, an miRNA—mRNA network.

Networks are built with two components: nodes, the participatory members; and edges, the connections between nodes, representing how they interact with each other [20]. When understanding and building miRNA networks, nodes can be of only one species of molecules; miRNAs, for example, resulting in monopartite graphs. Bipartite graphs, on the other hand, are built with nodes of two species of molecules [48]; miRNAs and molecules that they interact with, like mRNAs (Figure 1). Considering the complexity of biological systems, graphs should ideally be multipartite, with more than two species of molecules representing nodes. This approach would most likely build a complete network, with nodes being represented by miRNAs, mRNAs, other species of ncRNAs, pseudogenes, proteins, transcription factors and so forth. Unfortunately, thorough knowledge on all the players of the miRNA regulatory pathways remains to be discovered. The two possible and most feasible approaches, therefore, are constructing monopartite and bipartite networks. Each approach has its advantages and disadvantages, and different methods can be used to build each type. Thus, in this section, we review methods on how to build monopartite, association and bipartite networks and discuss the pros and cons of these approaches. Additionally, in Table 2 we include all the useful software and webtools to generate the presented networks.

Monopartite networks contain only miRNA nodes and are made using the expression level of miRNAs. This approach is abstract and reductive, since it considers the interaction patterns of miRNA species only. The main advantage is that it allows us to build separate networks for different patient cohorts (i.e., a network for the healthy group and a network for the pathological group), and subsequently enables a comparison of network architecture and structure between the cohorts [50]. The second advantage is that miRNAs that are not differentially expressed between patient cohorts can be included in the network analysis. Surprisingly, without having differences in expression between cohorts, some miRNAs have different connectivity inside the control versus pathologic miRNA network, a potential sign that they could play key roles [50]. A disadvantage of this approach is the fact that the meaning of an edge is unclear; the biological meaning of the connection between two miRNAs remains unknown and needs to be explored experimentally.

There are several methods to create this type of graph: (i) correlation coefficient method [59]; (ii) hierarchical cluster analysis [50]; and (iii) Bayesian network inference [60]. The correlation coefficient method is the simplest and most commonly used to build molecular networks, since it does not require the use of complicated algorithms or software. Therefore, this method will be presented in detail (Figure 1a). Protein-protein interactions and other types of biological networks were previously built using this approach [49,61]. Briefly, a correlation matrix with all the correlation coefficients (*r*) of all molecules is built using the expression level of several miRNAs from several patients from the same cohort. The entire method depends on the selection of a correlation threshold. In our previous publication, we used a very high threshold—*r* ≥ ±0.8, which is much higher than most correlations observed in biology, which are often between 0.3–0.4 [50]. In this case, a commonly used lower threshold would have made the network difficult to analyze because of the high number of edges. An alternative method of choosing a threshold is using the statistically significant correlation *p*-value—*p* < 0.05. The correlation *p*-value depends on the number of patient samples; therefore, a statistically significant *p*-value will correspond to a low *r* if the patient cohort is large, or to a high *r* if the cohort is small. The most important aspect that needs to be taken in consideration when choosing a threshold is that it should be the same for all the generated networks which will subsequently be compared (e.g., control network versus patient network). After choosing a threshold, interactions equal or higher than this value are searched for inside the correlation matrix (elements of the matrix that correlate equally to or are above the chosen threshold are considered connected nodes in the network). If the correlation of two miRNAs is equal or above the threshold, the correlation will be considered an edge between the equivalent miRNA nodes. Thus, from a statistical point of view, an edge represents a high correlation between two miRNAs; from a biological perspective, an edge remains a molecular interaction that needs to be experimentally validated. We have observed that miRNAs originating from the same miRNA family tend to correlate and create network motifs (patterns of interaction between elements of a graph that do not occur by chance). We hypothesize that an edge could represent a transcription factor that regulates the expression of these miRNAs or a molecule that sponges a group of miRNAs. Furthermore, we observed that viral miRNAs have a high tendency to correlate and build network motifs: the two KSHV miRNAs we analyzed (KSHV-K12-12* and KSHV-K12-10b) correlate and build an external network not connected to the main graph [50]. This can be intuitively explained by the fact that viral miRNAs are not codified by the human genome. In conclusion, this approach does not offer direct answers; however, it opens questions leading to the formulation of interesting hypotheses. The latter, importantly, must be confirmed experimentally at the bench.

The next step is detecting the hubs (most connected nodes) in the network and determining if in both pathological and normal conditions the hubs are the same. Extrapolating from computer science, the hub is the most vulnerable point of a network [44]. So, if an miRNA is a hub in the diseased patients’ network and an isolated or weakly connected element in the healthy cohort, it is fair to hypothesize that this could be a potential therapeutic target. This is a strong approach, since it does not only rely on the difference of expression between the groups. 

In order to strengthen the power of the results obtained, it is recommended to use two extra steps: (i) the use of expression data of miRNAs obtained by two different experimental methods (such as microarray or real-time qRT-PCR) and build multiple networks for the same patient cohort and compare the results—these should mostly overlap; and (ii) the use of two statistical methods (e.g., correlation coefficient and Bayesian inference) to build multiple networks for the same patient group and compare the results—again, these should partially overlap. 

Another method of building networks reemploys statistics and is based on association indexes, this method being a sort of hybrid between the mono- and bipartite approaches (Figure 1b). Herein, an edge between two miRNAs means the number of shared targets (usually mRNAs, because of the available data). There are four statistical association indexes that are frequently used: Simpson, Jaccard, Geometric and Cosine (for more details, see review by [48]). This method transforms a bipartite graph of miRNAs and mRNAs in a monopartite graph of miRNAs. Also, for this method, only highly expressed miRNAs are used to build the network, since they share targets and indirectly interact. Similar to the case of monopartite graphs, also these networks are based on thresholds, which represent levels of association. A threshold value of the association index is used to determine where an edge should be drawn. Hence, the meaning of an edge is a specific number of shared targets. From a strict biological point of view, two miRNAs sharing multiple targets could mean a common contribution to one or more signaling pathways that can be synergistic or additive. Two or more miRNAs can act in synergism to regulate a common mRNA, when both are needed to execute their function [62]; or their function can be additive, when only one miRNA is enough to regulate an mRNA, but, if acting together, the regulation is augmented [63].

Bipartite networks are composed of two types of nodes—besides miRNAs, other molecules that interact with them can be studied (Figure 1c). For the study of miRNAs, mRNAs are the most suitable partners in a network, because miRNAs regulate mRNA expression [15]. This approach is from the start more biological, since an edge has a clear meaning—an miRNA regulating an mRNA. Because this is known, the expression of an miRNA and its mRNA target usually negatively correlate; thus, when adopting this approach, only the differentially expressed miRNAs are selected to build networks. This converges to two main limitations: miRNAs that are stable between the control group and patient cohort are excluded, although their function could be important for cancer cells; and only one miRNA network that corresponds to the pathological state is built. Naturally, a second network can be built using the miRNAs that are overexpressed exclusively in the physiological state. However, the two networks will not have miRNA nodes in common, so no comparison between networks is possible. 

There are two different methods to build bipartite miRNA-mRNA graphs: (i) the experimental approach, where the expression of multiple miRNAs and mRNAs is determined and correlations are searched for (ideal miRNA-mRNA network); and (ii) based on published and validated literature and databases, like the Ingenuity Pathway Analysis (IPA) [58]. 

The first method used to build miRNA-mRNA networks performs a correlation analysis with the expression data experimentally measured (Figure 1(c.1)). A correlation matrix is built, similar to the one constructed for monopartite miRNA networks, except for the fact that rows will contain mRNAs and the columns, miRNAs. In this matrix, negative correlations will be searched for [57]. Also, the biological meaning of an up-regulated miRNA—down-regulated mRNA is easily interpretable: the miRNA controls the expression of the mRNA. On the other hand, the relationship between a down-regulated miRNA—up-regulated mRNA is not so explicit, since there are several mechanisms to increase mRNA levels other than miRNA post-transcriptional regulation. Another limitation of this approach is the dimension of the network. A network is built minimally with two nodes connected by an edge. Most of the networks built using this approach are not much bigger—it is resourceful and time consuming to measure the expression of hundreds of miRNAs and their several thousand corresponding targets. Thus, miRNA-mRNA interactions are usually presented by fragments of networks, with just one or two targets being regulated by a specific miRNA, like the case of miR-34, for example. It was reported that miR-34 regulates several targets in pathways involving apoptosis, genetic stress, EMT and the immune response (reviewed by [64]). The way these pathways were presented showed just a snap-shot in a most likely much bigger regulatory network involving miR-34. How these pathways interplay, or the complete regulatory network behind miR-34 function remains to be described. This method provides a small fragment of the molecular pathways and mechanisms controlled by the miRNAs studied, but it does not identify all the possible regulatory molecules that could be involved with the miRNAs of interest. 

The second and most commonly used method to build any type of miRNA network is the literature research strategy, manually curated, or by using different software, such as IPA (Figure 1(c.2)). Manually curated miRNA–mRNA interactions can be retrieved by using available miRNA–target databases [miRTarBase (available online: http://mirtarbase.mbc.nctu.edu.tw/php/index.php [65]), DIANA tools—TarBase (available online: http://diana.imis.athena-innovation.gr/DianaTools/index.php?r=tarbase/index [66]), miRWalk (available online: http://zmf.umm.uni-heidelberg.de/apps/zmf/mirwalk2/ [67]), miRecords (available online: http://c1.accurascience.com/miRecords/ [68])]. In order to generate the miRNA-mRNA bipartite networks, experimentally validated targets and predicted targets can be chosen. Because most miRNAs have hundreds of validated targets and thousands of predicted targets, researchers should chose inclusion criteria for the interactions of interest (e.g., miRNA-mRNA interactions which are validated by strong methods, showing a functional phenotype or miRNA-mRNA interactions that are appropriate for the study’s context, in this case cancer). On the other hand, IPA uses a causal network approach and generates networks based on prior published biological data [58,69,70,71,72,73]. The whole construct is actually based on a Knowledge Base, a huge systematized collection of biological data. The Ingenuity Knowledge Base is a network built out of 40,000 nodes, including mostly miRNAs, and approximately 1,480,000 edges. By imputing the differentially expressed miRNAs, the IPA software calculates, by applying an enrichment score and a *Z-score*, which are the most probable upstream or downstream interactors [58]. The first limitation of this method is that it is based on a biological database that contains information from numerous physiological and pathological states and information from different cell and tissue types. That raises the question if such a diverse *a priori* biological information could be extrapolated to a given particular case. Secondly, the entire IPA network construct does not take into account the individual expression levels of the studied molecules, everything being described as on or off. The advantage of literature research networks is that they are real biological networks based on experimental data. Additionally, these networks are very popular because they are easy to use and to understand. 

**Figure 2:**
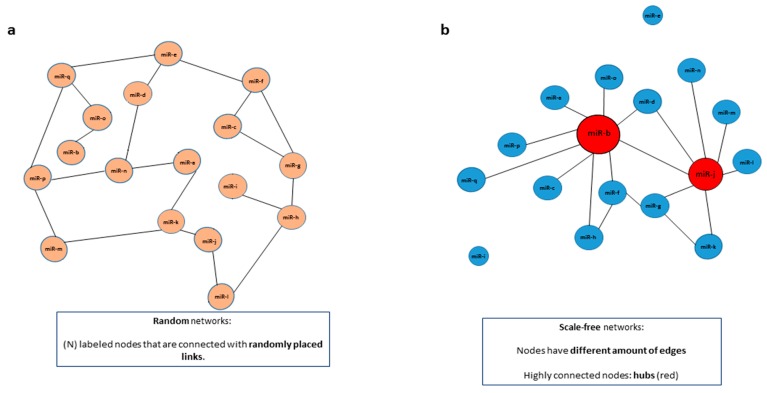
Networks can be characterized as random or scale-free. Networks can be classified as random or scale-free, depending on how the edges are shared among the nodes. (**a**) In random networks, (N) labeled nodes are connected with randomly distributed links (edges). (**b**) In scale-free networks, on the other hand, nodes have a different amount of edges and are not distributed randomly. In a scale-free miRNA network some miRNAs (in red) have more connections than others (in blue). Highly connected nodes, in this case represented by miRNAs in red, are termed hubs.

Additionally, networks can be characterized as being random or scale-free. In random networks, N labeled nodes are connected with L randomly distributed links (edges) [74] (Figure 2a). Scale-free networks represent nodes that differ significantly in the number of connections, meaning that certain nodes share more edges than others [20,47] (Figure 2b). These highly connected nodes (hubs), are usually more representative of biological networks, since some genes (i.e., miRNAs) are essentially more important than others, depending on the context. In fact, pathways involving network hubs are sometimes more enriched in biological processes, including in cancer. These constant clusters of interactions are termed network motifs. Common biological processes that represent network motifs are, for example, feedback and feedforward loops and control of gene expression through competitive interactions [20]. Several of the most important miRNAs discovered to date to be strongly involved in cancer take part in these pathways, like the miR-17-92 cluster participating in the regulatory network of MYC [75]. Perhaps comprehending as much as possible the edges and nodes involved in these motifs could direct researchers into more powerful therapeutic approaches for miRNAs in cancer. 

## 3. Examples of miRNA Network Analyses

The study by Piepoli and colleagues, building monopartite graphs using Pearson’s correlation coefficient method, identified miRNAs to be potential drivers of colorectal and pancreatic cancer [59]. Tissue-specific patterns of miRNA deregulation were traced by the network analysis: the driving miRNAs were miR-195, miR-1280, miR-140-3p and miR-1246 in colorectal tumors, and miR-103, miR-23a and miR-15b in pancreatic cancers. A limitation of this study is that the network was built upon deregulated miRNA expression alone. There was no analysis about their possible targets or on the miRNAs that are not differentially expressed that could be participating in a network. Still, it represents an important section of miRNA regulatory networks, and a significant start in understanding their roles in different pathways.

An interesting study published in 2010 revealed that miRNA networks are reprogrammed in cancer [60]. The study was based on expression data of healthy donors and patient samples, the latter including 51 types of cancer, both from solid tumors and leukemia. The microarray data from over 3000 patients were processed by the Bayesian network inference method, to build monopartite graphs, stating the connection between miRNAs in different types of cancers. It was showed that, not only the pattern of expression of the miRNAs change, but also the connection between them, when comparing normal versus tumor samples. The authors described a redesign of miRNA networks in cancer. Unfortunately, this approach does not take into account other molecules involved in miRNA function and biology. It would be more challenging to develop efficient therapies based solely on miRNA expression and interaction.

Bipartite graphs will bring more complex networks, since they take into consideration the relationship of miRNAs and their targets, or other molecules involved in miRNA function. The most frequent method to build bipartite networks is using databases of a priori available biological data. One of the most commonly used tools to infer relationship between miRNAs and their respective targets is the commercially available software IPA, based on published and validated literature and databases. The greatest advantage of this approach is how easy the interface between user and software is. Also, several patterns of interactions are conserved between tissues and types of cancer, in this case, making it easier to identify upstream biological causes and downstream consequences of dysregulations in miRNA networks. A downside of this method, however, is that some miRNA networks are so intricate and specific to that type of tissue in that type of cancer, that the IPA analysis can lead to wrong causal inferences in a particular network. In some cases, miRNAs can act as oncomiRs or tumor suppressors, depending on the tissue and type of cancer [41]. For example, miR-125b acts as an oncogene in the majority of hematological malignancies [76], while as a tumor suppressor in many solid tumors [77]; or miR-155, largely considered an oncomiR in many types of cancers [78,79,80], but already proven to act as a tumor suppressor in melanoma, gastric and ovarian cancer, and acute myeloid leukemia (AML) [78,81,82,83]. Therefore, one should be cautious about the inferences made based on connections observed in IPA analysis. Nevertheless, studies of miRNA targets and their functions were developed using the IPA analysis as a start. For example, the identification of miR-21 as being one of the key players of tumorigenesis in glioblastoma, and how it mainly acts by the identification of its targets [84]. Importantly, these targets turned out to be present in key tumor suppressive pathways of glioblastoma, making miR-21 a hot target for development of therapeutic approaches and possibly as a diagnostic tool. 

A study published in 2015 presented miR-940 as being a strong target for breast cancer therapy, based on a network built with common miRNA targets [85]. The authors used the association index method to build a target-based network, considering the fact that if two miRNAs share a set of genes, they could possibly be involved in the same pathways. They evaluated the strength of an edge, the connection between two miRNAs, by a statistical method, the Simpson index. The authors showed that miR-940 is essential in breast cancer, a fact that was never proved using only expression analysis. Using the network analysis results, the authors demonstrated that miR-940 is important for cytoskeleton regulation and cell movement, by experimental approaches, describing a possible new strong miRNA target in breast cancer. The fact that this approach used the connection between their targets, instead of miRNA expression alone, makes these data more robust. By identifying common targets, the authors speculated that related miRNAs could be compensating each other or act complementarily in the same pathways. A few networks were presented, and miR-940 was concluded to play a key role in breast cancer metastasis. This approach increased the complexity of the network, bringing it closer to biological systems.

Transcriptome-wide studies focus not only on miRNAs and their targets, but also on all possible molecules involved in miRNA regulation and function. These studies come closer to an ideal miRNA-mRNA network, since they are based on large sets of experimentally validated data. A study published in 2014 developed a method to search for and analyze several components of ncRNA pathways, based on basically ncRNA-mRNA and ncRNA-protein binding [86]. Among the ncRNA species analyzed were miRNAs, lncRNAs and circRNAs, besides pseudogenes and RNA-binding proteins (RBPs). The experimental technique, Cross-linking Immunoprecipitation followed by deep sequencing (CLIP-Seq), has successfully and reliably identified Argonaute (Ago) and other RBP binding sites. By identifying millions of Ago and the most common RBP binding sites, the group built networks of interaction between several classes of molecules based on their ability to interact with each other. The approach is interesting, since it comprises several molecules involved in gene expression and ncRNA function and their respective targets. 

There are several ways with which miRNA interaction networks can be built, in order to study their regulatory functions. The best method of analysis is not well defined, since the interactions that could be discovered need to be validated experimentally. Ideally, miRNA regulatory networks should be predicted as complete as possible, with all classes of ncRNAs and proteins that could be involved. The greatest challenge remains to validate these interactions at the bench. 

## 4. Therapeutic Perspectives of miRNA Networks in Cancer

Since miRNAs are molecules that participate in numerous regulatory pathways in a cell, it is important to understand with whom and how these interactions occur in order to consider miRNAs as therapeutic approaches. 

Single-targeting of miRNAs or non-hub elements (marginal nodes) in an miRNA network has proven to be inefficient in in vitro and in vivo studies, as well as in the clinic. In fact, as mentioned before, a promising therapeutic strategy involving miR-34 for treatment of liver cancer and liver metastasis failed when applied in the clinic. Unfortunately, some patients that received the treatment had severe side effects, while others were deceased [40]. In these cases, it is hard to predict and evaluate the cause that led to those particular side effects or death, especially because of all the interactions with which miR-34 might participate in and the molecules involved are still not completely known. It is coherent to speculate that higher concentrations of miR-34 in the cell or in the blood could have triggered pathways that culminated in the side effects reported or even death. Knowing the in vivo toxicity of miRNA therapy is not enough to proceed to clinical trials. We must understand the entire regulatory network to predict the enhancement or shutting down of vital pathways. 

The efficacy of a single miRNA therapy can also be questioned. Mechanisms of drug resistance and tumor recurrence can and probably will occur due to compensatory pathways present in the same network (reviewed by [87]). Thus, aiming at marginal nodes might lead to a deviation of a pathway in a certain network, but the outcome might still be the same through alternative routes. There have been several observational studies on patients with various types of cancer to identify the expression pattern of miRNAs before and after receiving a given therapy (reviewed by [88]).

Targeting network hubs seems like a more successful approach. However, since miRNA are highly interconnected, aiming at hubs might have catastrophic consequences, because these elements might confer stability and robustness to a network [44,89]. Therefore, one should be extremely careful in designing and analyzing miRNA networks when defining therapeutic targets.

It is important to assess the differences between miRNA network patterns in physiological versus malignant cells. For example, a comparison of miRNA regulatory networks in normal tissues against solid tumors and leukemia showed differences between hubs and in the network architecture [60]. Identifying these patterns in cancer might be more successful in delineating network-based therapeutic approaches. Also, important to emphasize here are recent studies demonstrating that the gene expression pattern does not entirely represent the robustness of cellular pathways. When trying to identify essential genes, some groups have developed CRISPR (Clustered Regularly Interspaced Short Palindromic Repeats) knockout library screens of several cell lines [90,91]. Besides being able to identify specific genes, it was found that the correlated genetic interaction profile between genes (or their respective end-product, may be a protein or an ncRNA, for example) proved to be more relevant in determining their essentiality for cell survival than their expression level. That is because, when identifying or understanding this profile, there is a higher chance that the correlated genes are involved in the same pathways. Dissecting the pathways and understanding how their molecules participate in them could lead to the identification of a drug targetable network to treat cancer, for example, especially and essentially in the case of miRNAs, which target hundreds of molecules of the cellular regulatory network.

Despite cancer treatment, miRNA networks could and should also be used for the design of diagnostic approaches. Since differences in miRNA network patterns have been detected in healthy versus tumor cells, it is possible that cancer patients could have an miRNA network signature that could be detected and used for diagnosis. Even more specifically, miRNA network patterns could be used as prognostic factors, considering the fact that cancer patients could present different signatures depending on the stage of the disease. These considerations should be taken into account when using miRNAs for the development of diagnostic tools, since, as extensively stated, the pattern of molecular interactions of miRNAs is extremely intricate. 

Finally, it is crucial to understand that building comprehensive miRNA network analyses facilitates the development of anticancer strategies, especially of multi-targeted and combinatorial therapies that are already employed in the clinic, such as chemotherapy. Overall, miRNA networks should be a starting point on trying to understand their roles in tumorigenesis and disease progression, given their extensively discussed complexity. Nevertheless, organismal biology has proven to be just as complex, and validation through experimental data must be assessed before any progression of miRNA-based therapy in the clinic.

## Figures and Tables

**Table 1:** Definitions of network components and types of networks.

Basic Nomenclature	Definitions
Nodes	Elements of a network.
Edges	Interactions/connections between elements of a network.
Hubs	Nodes with a high degree of interactions.
Association index	Method used for the quantification of similarity between nodes of the same species. The most common used association indices are: Jaccard, Simpson, Geometric and Cosin.
Monopartite network	Graph that contains only one species of nodes, such as a miRNA network.
Bipartite network	Graph that contains two species, with edges representing their interaction pattern, such as miRNA-mRNA networks.
Multipartite network	Graph that contains more than two species of nodes.
Association networks	Networks in which two nodes of the same type are connected only if their similarity calculated using an *association index* is above a selected threshold.
Scale free network	A network that follows a power-law distribution, with some nodes that have a higher number of connections (*hubs*).
Random network	A network with (N) labeled nodes that are connected with randomly distributed links (edges).
Undirected network	A graph in which the edges between nodes have no orientation.
Directed network	A graph in which edges between nodes have orientation. An edge is depicted as an arrow, is directed from node a to node b and is not equivalent to an edge from node b to node a.

**Table 2:** Useful software and webtools to generate monopartite, association and bipartite networks.

Network Type	Method	Examples of Software/Webtools	Reference
Monopartite network	Correlation coefficient	Any statistics analysis software (GraphPad Prism, IBM SPSS, R)	[49,50]
	Hierarchical clustering	IBM SPSS, R	[50,51,52] *
	Bayesian inference	Banjo (Bayesian network inference with Java objects) https://users.cs.duke.edu/~amink/software/banjo/	[53,54,55]
Association networks	Association indexes	GAIN http://csbio.cs.umn.edu/similarity_index/login.php	[48]
Bipartite networks	Experimental approach (correlation coefficient)	Any statistics analysis software (GraphPad Prism, IBM SPSS, R)	[56,57]
	Automatic literature search	IPA Qiagen	[58]

* Reference [50] presents how to use the IBM SPSS software to build an miRNA hierarchical cluster network, and references [51,52] describe theoretically how hierarchical clustering can be used to generate networks, without referring to specific software.

## Abbreviations

miRNAs	MicroRNAs
ncRNAs	Non-coding RNAs
Lin-4	Lineage defective 4
Let-7	Lethal 7
mRNAs	Messenger RNAs
circRNAs	Circular RNAs
lncRNAs	Long non-coding RNAs
OncomiRs	Oncogenic microRNAs
CLL	Chronic Lymphocytic Leukemia
AML	Acute myeloid leukemia
ceRNAs	Competitive endogenous RNAs
MREs	MiRNA response elements
EBV	Epstein-Barr virus
EMT	Epithelial-to-mesenchymal transition
PD-L1	Programmed death-ligand 1
MUC1	Mucin 1
Mimics	Mimic endogenous microRNAs
AntagomiRs	MicroRNA inhibitors
ceRNAs	Competitive endogenous microRNAs
KSHV	Kaposi’s sarcoma-associated herpesvirus
Real-time qRT-PCR	Real-time reverse transcription polymerase chain reaction
IPA	Ingenuity Pathway Analysis
RBPs	RNA-binding proteins
CLIP-seq	Cross-linking Immunoprecipitation followed by deep sequencing
Ago	Argonaute
CRISPR	Clustered Regularly Interspaced Short Palindromic Repeats
